# Randomized control trial of a childhood obesity prevention family-based program: “Abriendo Caminos” and effects on BMI

**DOI:** 10.3389/fped.2023.1137825

**Published:** 2023-06-07

**Authors:** Margarita Teran-Garcia, Amber J. Hammons, Norma Olvera, Kimberly Greder, Maria Plaza-Delrestre, Flavia Cristina Drumond Andrade, Barbara Fiese, Angela R. Wiley

**Affiliations:** ^1^ University of Illinois Extension, Division of Nutritional Sciences, Family Resilience Center, College of Agricultural, Consumer and Environmental Sciences, Carle Illinois College of Medicine, University of Illinois Urbana-Champaign, Champaign, IL, United States; ^2^ Department of Child and Family Science, California State University, Fresno, Fresno, CA, United States; ^3^ Psychological, Health, and Learning Sciences Department, University of Houston, Houston, TX, United States; ^4^ Human Development and Family Studies, Iowa State University, Ames, IA, United States; ^5^ Agro-Environmental Science Department, Food Science and Technology Program, University of Puerto Rico, Mayagüez, Puerto Rico; ^6^ School of Social Work, University of Illinois Urbana-Champaign, Urbana, IL, United States; ^7^ Department of Human Development and Family Studies, Family Resilience Center, College of Agricultural, Consumer and Environmental Sciences, University of Illinois Urbana-Champaign, Champaign, IL, United States

**Keywords:** childhood obesity, obesity prevention, Hispanics, family-based program, BMI z-score

## Abstract

**Background:**

Hispanic individuals are at increased risk for obesity and other chronic health conditions. This article evaluates the effect of a family-based, childhood obesity primary prevention intervention in a community setting.

**Methods:**

A multi-site, randomized controlled trial community program with assessments at pre (T0), post-program (T1), and 6-months post-program (T2). Participating families were recruited from five sites. Only families of Mexican or Puerto Rican heritage with a least one child between 6 and 18 years were included in the study, without weight restrictions. Families were randomized to the intervention and control arms. Intervention families received six-2 h weekly workshops. Control families received printed generic nutrition and wellness information. Heights and weights were measured at the 3-time points to calculate BMI z-scores, BMI-percentiles, and weight status using age- and sex-specific growth charts, according to the CDC guidelines.

**Results:**

There were no differences in BMI-z scores between children in the intervention (*n* = 239) and control groups (*n* = 187) at T0. BMI z-scores decreased in the intervention group (−0.03, 95% CI, −0.066, −0.003, *p* = 0.032) at T1, but not in the control group at T1. Changes in BMI z-scores were not statistically significant at T2.

**Conclusion:**

The Abriendo Caminos intervention effectively prevented unhealthy weight gain in Hispanic children in the short-term, but not at 6-months post-intervention. Younger children and girls benefited more from the program at 6-months post-intervention. Additional efforts are needed to sustain long-term changes. Culturally-tailored programs can provide families with the knowledge to produce short-term changes and a potential pathway for sustainable changes in implementing healthy behaviors.

## 1. Introduction

Hispanic-heritage individuals are profoundly affected by chronic metabolic conditions (CMCs: i.e., obesity, diabetes, dyslipidemia, and hypertension), and inequities in the prevention and management of those diseases severely affect their health and quality of (1–4). In adults, the prevalence of CMCs is higher among Hispanics than in non-Hispanic white people (5, 6). In addition to CMCs, behavioral factors, such as physical inactivity and smoking, represent the primary clinical risk factors related to cardiovascular heart disease, the leading cause of death in the U.S. (1). Hispanic children (ages 6–11) and adolescents (ages 12–19) also have higher levels of obesity and severe obesity relative to non-Hispanic white people (7). Children affected with obesity often become obese as adults and have an increased risk for a wide variety of poor health outcomes, including CMCs, and nonalcoholic fatty liver (8, 9). Additionally, children are at higher risk of developing weight problems when they start formal education, as they exert increasingly independent eating choices and engage in diverse levels of physical activity (10). There is a critical need to develop primary and secondary childhood obesity prevention programs.

Measuring the effectiveness of many childhood obesity primary prevention interventions is limited by indirect and self-reported behavioral changes. Alternatively, some primary prevention programs with growing children aim to reduce or maintain sex and age-specific BMI percentiles, or maintain a stable weight over one year (11, 12). In practice, BMI measurements adjusted for child age and sex are converted to standard deviation scores (SDS), also called BMI z-scores, to measure the variation around the mean (SDS equal to 0), and a negative BMI-SDS indicates the value is below the average. Most researchers report that a decrease in BMI-SDS greater than 0.5 is associated with success in weight management interventions or a reduction in CMCs (11–13). The −0.5 BMI-SDS value is equivalent to a child with a stable weight growing 5 cm or more per year, or a 5 kg weight loss in a male adolescent with 1 cm growth in 12 months (14). Even a BMI-SDS reduction of 0.25 or greater has been found to significantly improve cardiovascular risk factors (hypertension, hypertriglyceridemia, and low HDL-cholesterol) in children with overweight followed over a 1-year lifestyle intervention, while a BMI-SDS decrease greater than 0.5 doubled the effect (11).

Primary and secondary obesity prevention interventions with children are more likely to be successful when they include a parental or family component and target multiple health behaviors (diet, physical activity, sleep) (15–21). Recent reviews underline the impact of evidence-based and family-based lifestyle preventive interventions that target multiple modifiable lifestyle behaviors (16, 18, 22). Still, there are few culturally-tailored, family-based programs demonstrating improving health-related outcomes directed to Hispanics (12, 15, 16, 20). A systematic review of nine obesity interventions with Hispanic children conducted between 2000 and 2010 found that only four studies had significant changes in BMI, and all four included a parental component (18). The authors noted that few studies included follow-up evaluations with their participants, which is important in assessing the sustainability of changes. Two recent reviews focused on Hispanic family-centered interventions found similar findings, with approximately a third of the studies reporting significant changes in BMI (11, 20). Reviewers suggested that culturally relevant family-based programs are essential in controlling obesity in Hispanic families and should incorporate a focus on diet and exercise, as well as sleep and screen time, to improve effectiveness (11, 20). According to a family systems approach, the entire nuclear *family* should be included as families are interconnected and influence one another's behaviors (23). Additionally, evidence-based interventions are more likely to be effective when they have a theoretical framework and are delivered by a dedicated staff (24, 25). Taken together, obesity programs seem more effective at reducing BMI when they are culturally tailored, grounded in theory, include the entire family, target multiple healthy behaviors, and are long-term.

*Abriendo Caminos,* “opening roads”, is designed as an evidence-based, theoretically grounded, culturally tailored, primary childhood obesity prevention program, and implemented as a parallel-arm, randomized control intervention (22). *Abriendo Caminos* is grounded in the applied behavior theory for community nutrition programs and tenets of the social cognitive theory (22). The intervention was implemented across five sites in California, Illinois, Iowa, Texas, and Puerto Rico. According to the latest data, the obesity percentiles in the participating states are 47th, 23rd, 11th, and 13th respectively, but no comparative obesity prevalence data exist for the Commonwealth of Puerto (26). The 2021–2022 National Survey of Children's Health reports that 34.7%, 32.4%, 34.5%, 39.5% of children and adolescents (ages 10–17) from California, Illinois, Iowa, and Texas are affected by overweight or obesity. Furthermore, in California, Illinois, Iowa, and Texas, 44.3%, 49.2%, 43.2%, and 49.2% of Hispanic children and adolescents of the same age group are affected by overweight or obesity, underlying a health disparity (26). Puerto Rico's rates of childhood obesity are estimated to be between 28% to 30%.

The current study aimed to assess the impact of the *Abriendo Caminos* intervention on the BMI z-scores outcome of children and adolescents. The community program incorporates multiple elements to prevent unhealthy weight gain, with a whole family approach focusing on nutrition education, family wellness, and physical activity (22). It was hypothesized that the *Abriendo Caminos* program would be effective if the intervention group had no significant BMI z-score change, or if participants maintained a stable BMI z-score over the 6 months of observation compared to the control group.

## 2. Methods

### 2.1. Study design

The research methods have been described elsewhere (22). *Abriendo Caminos* is a multi-site, longitudinal, randomized control obesity primary prevention intervention designed to reduce excessive weight gain in Mexican and Puerto Rican children in a community setting. Randomization into the intervention and control groups was conducted during initial data collection for each cycle in each cohort. Randomization process was determined by site, and participating families were randomized using a random number generator (Research Randomizer, Lancaster, PA, 2013), or by participants selecting a ball from a bag, whose color corresponded to a specific treatment arm. Families were randomized in a ratio of 1:1 per cycle of the program. Groups were not matched on child age or sex. The number of families and participants in each cohort varied depending on staffing and space capacity at each site and the number of eligible families available at each time. On average, 12–20 families participated per cohort. The *Abriendo Caminos* intervention was available in Spanish and English; it was delivered by dedicated and trained staff. Each site had a primary investigator, who recruited bilingual staff, including graduate and undergraduate pre-health, human nutrition, human development, community health, or kinesiology students, and -when possible- extension and volunteer personnel embedded in the local community. All personnel who delivered the program participated in extensive training (about 20 h, under the “train the trainer” model) and had weekly debriefs during and after program implementation.

### 2.2. Study sites and recruitment

The selected sites in Illinois (Champaign, Urbana, and Rantoul), and Iowa (Ottumwa and Perry), are suburban, while those in Texas, California, and Puerto Rico are urban. The Illinois and Iowa sites were associated with well-established University Outreach /Cooperative Extension/Community collaboration. At the Texas site (Houston), Hispanic families were recruited from an urban Houston Metropolitan area targeting a low-income, predominantly Mexican neighborhood named East End. Due to the long relationship with the community, potential Hispanic families were recruited from social services agencies such as community centers and clinics. In California, the site (Fresno) is a major city in the Central Valley. Project coordinators and research assistants contacted families in multiple ways, including passing out flyers at Hispanic grocery stores, flea markets, elementary schools, and churches. At the Puerto Rico site, families were recruited from the west side of the island. This was due to the location of the University of Puerto Rico at Mayaguez (UPRM). The University of Puerto Rico, Mayagüez Campus, is a public land-grant university. UPRM is the second-largest university campus of the University of Puerto Rico system. In addition to its status as a land-grant university, it is also a member of the sea-grant and space-grant research. More information is described in detail at the clinical trial registration.

### 2.3. Participants

Mexican and Puerto Rican descent families enrolled in this study (at least one parent and one child aged 6–18 years per family). There were no upper or lower weight restrictions for children to participate in the community program. Participants were recruited using stakeholder engagement, local newspaper advertisements, and flyers/cards displayed and passed out at recreation centers, grocery stores, flea markets, churches, and local Cooperative Extension offices. Interested participants were asked to attend a study orientation or provide contact information and/or contact the local site coordinator(s) to determine study eligibility. This study adhered to the CONSORT statement reporting guidelines and was registered with www.ClinicalTrials.gov (NCT03505658). Before baseline data collection, bilingual research assistants asked parents to read and sign the informed consent and target children were asked to sign an assent form, respectively, confirming that they understood the terms of participation. Families were informed that their participation in the study was voluntary, and they were permitted to withdraw at any point without explanation. Due to the US political climate at the time of the study initiation, 2016, we secured a "certificate of confidentiality" and removed participant names from the data.

A sample size of 500 families (250 intervention and 250 control) was pre-planned to provide adequate power to detect the intervention effects based on the original project (22). A staggered initiation of sites was designed to implement regional adaptations of the project. Thus, the first site was Illinois, and the second and third were California and Iowa. Finally, for the fourth and fifth sites, we encountered multiple natural disaster challenges, including weathering a major earthquake and three hurricanes (Harvey, Maria, and Irma), delaying data collection in Texas and Puerto Rico. Moreover, the COVID-19 pandemic presented unprecedented and unpredictable challenges in recruiting, implementing, and evaluating a face-to-face intervention. In the end, two sites had to adapt workshops and recruitment to a virtual delivery (Texas and Puerto Rico). For methodological consistency, only the data from participants who engaged in the in-person workshops and recruitment are presented here (*n* = 426).

### 2.4. Measures

Mostly mothers completed survey data and anthropometric assessments at pre-program at baseline (T0), after the 6-week intervention (T1, post-program), and six-months post-intervention (T2). Demographic data collected at T0 from mothers included birth country, child's date of birth, and child's sex. Trained research assistants measured children's body height and weight at least twice using Seca (Seca North America, Chino, CA) stadiometers and scales. Body height was measured in a standing position with both feet touching the base of the board and the head in the Frankfort Plane. Body weight was measured in light clothing. The average of the two measurements was recorded. If the difference between the two measurements was greater than 0.1 kg or 1 cm, a third measurement was taken. BMI was calculated using kg/m^2^ formula and transformed into age- and sex-specific z-scores and percentiles according to the Centers for Disease Control and Prevention (CDC) growth charts (27). Child BMI weight status was determined using BMI percentiles, to classify as underweight (BMI percentile <5th), healthy or normal (BMI percentile 5th to <85th), overweight (BMI percentile 85th to <95th), and obese (BMI percentile ≥95th) (27).

#### 2.4.1. Experimental group

As described elsewhere, the Abriendo Caminos program is educational, with 6 weekly 2-hour workshops (22). Abriendo Caminos is a community-based delivery program with cultural-tailoring. Briefly, the applied behavior theory for community nutrition with the tenets of Social Cognitive Theory (behavioral capacity, self-efficacy, and social support) is integral to the curriculum to facilitate behavioral change. We used a simple Spanish linguistic structure with the concept “*más*,” “*menos*,” or “*más o menos*” (more, less, or in between) integrated into lessons to focus on small changes to increase healthier lifestyles and reduce negative or restrictive behaviors (22). The intervention program included workshops with three components (30 min of nutrition education, 30 min of family wellness education, and 60 min of physical activity), with extensive details published before (19, 22, 28). At the end of each session, parents completed an evaluation of the workshop and received a gift card for their attendance.

#### 2.4.2. Control group

Control families completed the demographic questionnaires and had their anthropometric measurements taken at T0, T1, and T2. Control group families did not participate in the workshops, but they received educational materials at the end of the 6-month period.

### 2.5. Statistical analyses

Descriptive statistics (means, standard deviations, median and interquartile range, and percentages) were used to compare characteristics of children in the intervention vs. the control groups. Group differences across intervention arms were examined using chi-square tests for categorical covariates and *t*-tests or Wilcoxon rank-sum tests for continuous variables.

**Figure 1:**
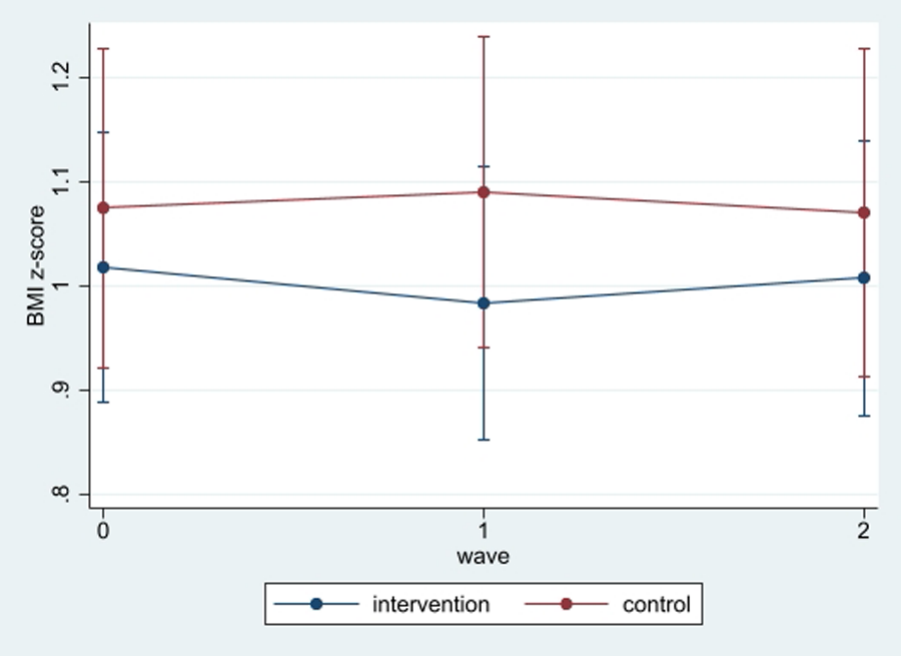
BMI z-scores changes during the study. At baseline, those in the control group had slightly higher BMI z-scores than those in the intervention group, but the difference was not statistically significant. After the first follow-up (T1), the intervention group experienced a reduction in BMI z-scores, relative to the baseline, but not the control group. At the end of the second follow-up, the intervention group maintained their improved scores compared to the first wave assessment. Still, the control group continued to observe increases in the scores. However, differences between the intervention and control group were not statistically significant at 6 months.

The primary outcome for this report was the change in BMI z-scores. We used a repeated mixed-effects linear regression (29, 30) to analyze longitudinally changes in BMI z-scores at 6-weeks post-intervention (T1), and 6-month post-intervention (T2) compared to the baseline values. Repeated mixed-effects regressions are appropriate for handling correlated data and an unequal number of observations across individuals, which is an advantage over generalized linear models. Measures of BMI z-scores were treated as continuous. The full model included: age, sex, intervention group, time of measurement (T1, T2), interaction of group and time of measurement, and intervention site. Random effects for the intercept were included to allow individuals to vary in the initial level of BMI z-score at baseline (T0). Additionally, analyses were disaggregated by sex and age group (6–11, and 12 or older). Finally, since most mothers were from Mexico, we restricted the analyses to those participants. We presented the regression coefficients, confidence intervals, and *p*-values. However, to facilitate the interpretation of regression results, particularly the interaction effects, we discussed the contrasts involving factor variables and their interactions using the “contrast” command.

Finally, we conducted sensitivity tests. First, we examined whether dropping out of the sample affected the results. To do so, a dichotomous variable was added to indicate those with complete data on all waves vs. those with missing data on at least one follow-up. Then, we included a dummy variable for incomplete vs. complete data in the mixed-effects. Lastly, we restricted data to those with complete data in all waves. Statistical significance was set at *p* < 0.05. All data analyses were conducted using the statistical software Stata/SE 17.0 (StataCorp, TX, USA).

## 3. Results

### 3.1. Baseline characteristics of the study sample

A total of 426 participants had complete data at the baseline, with 239 randomly assigned to the Abriendo Caminos intervention group and 187 to the control group. Of the 426 participants, 276 (65%) had complete data in the post-program (T1), 158 in the intervention group, and 118 in the control group. At the end of six-months (T2), 185 participants (43%) completed data collection—105 in the intervention group and 80 in the control group. The results showed that older children, 25% of the participants, were more likely to drop out than younger children (OR = 1.10, 95% CI, 1.02–1.18; *p* < 0.001). Groups were similar in the baseline with no significant group differences in demographic or anthropometric variables. Table 1 provides participant characteristics at baseline with stratification by intervention arm. Baseline characteristics include 56% girl participants, the average age was 10.2 years, mean BMI z-score was 1.04 and mean BMI percentile was 83.9. Over half of the participating children were affected by obesity or overweight (35.5% and 18.3%, respectively). Mothers were, on average, 39.4 years-old, 28.3% were overweight and 59.8% were affected by obesity. The majority of mothers were born in Mexico (94.7%), and most were married (79%). Over half had less than a high school education, and 31% lived in households with annual income below $30,000 US dollars. There were no statistical differences in children or mothers' demographic characteristics between the intervention and control groups at baseline.

**Table 1:** Descriptive statistics at the baseline of *Abriendo Caminos* participants.

Child characteristics	Total (*n* = 426)	Intervention (*n* = 239)	Control (*n* = 187)	*p-*value
Age, years (mean ± SD)	10.15 ± 2.87	9.98 ± 2.71	10.36 ± 3.07	n.s.
Ages 6–11 (%, *n*)	74.7% (318)	77.8% (186)	70.6% (132)	
Ages 12 or older (%, *n*)	25.4% (108)	22.2% (53)	29.4% (55)	
Sex (%)				n.s.
Boys, % (*n*)	44.75% (189)	43.10% (103)	45.99% (86)	
Girls, % (*n*)	55.25% (237)	56.90% (136)	54.01% (101)	
BMI z-score (mean ± SD)	1.04 ± 1.08	1.01 ± 1.08	1.07 ± 1.08	n.s.
BMI percentile (mean ± SD)	83.87 ± 35.09	83.22 ± 34.86	84.70 ± 35.47	n.s.
BMI percentile categories, % (*n*)				
Underweight (<5th)	1.64% (7)	2.09% (5)	1.07% (2)	
Normal weight (5th–85th)	44.60% (190)	46.44% (111)	42.25% (79)	
Overweight (85th–95th)	18.31% (78)	17.15% (41)	19.79% (37)	
Obese (>95th)	35.45% (151)	34.31% (82)	36.90% (69)	
Mother characteristics	(*n* = 430)	(*n* = 235)	(*n* = 195)	
Age, years (mean ± SD)	39.44 (8.63)	39.24 (8.05)	39.67 (9.29)	n.s.
BMI, kg/m^2^ (mean ± SD)	32.54 (7.10)	32.15 (7.38)	33.00 (6.74)	n.s.
Weight status^a^, % (*n*)				
Normal (18.5–24.9 kg/m^2^)	11.85% (52)	13.08% (31)	10.61% (21)	
Overweight (25.0–29.9 kg/m^2^)	28.32% (124)	30.38% (72)	26.26% (52)	
Obese (≥30 kg/m^2^)	59.84% (259)	56.54% (134)	63.13% (125)	
Place of birth, % (*n*)				
United States	3.64% (13)	3.40% (7)	3.87% (6)	
Mexico	94.7% (342)	95.15% (195)	94.19% (146)	
Other countries	1.71% (7)	1.47% (3)	1.94% (3)	
Marital status				
Married or Living with Partner	78.98% (294)	79.43% (166)	78.53% (128)	
Other	21.02% (78)	20.58% (43)	21.47% (35)	
Education, % (*n*)				
High School or More	46.75% (181)	46.98% (101)	46.51% (80)	n.s.
Annual income, % (*n*)				
$30,000 or more	31.27% (103)	31.44% (61)	31.11 (42)	n.s.
Less than $30,000	68.73% (226)	68.56% (133)	68.89% (93)	n.s.

BMI, Body mass index.

Data are mean ± standard deviation (SD), or % (*n*).

^a^ Based on the age and sex-specific CDC BMI growth charts.

### 3.2. Comparison of intervention groups across time

Table 2 shows the mean of the BMI z-scores over the study period. Mean BMI z-scores were 1.04 at baseline, 1.02 immediately after the 6-week program, and 1.11 at six-months after the end of the program. Mean BMI z-scores for the control group were 1.07 pre- and post-program and reached 1.10 at six-months. For the intervention group, at baseline mean BMI z-score was 1.01, reached 0.98 after completion of the 6-week program and 1.12 after six-months. Based on a simple T- test, there were no statistical differences between the control and intervention groups at each assessment time point (Figure 1).

**Table 2:** Mean BMI z-scores and 95% confidence intervals (CI) during the *Abriendo Caminos* intervention.

Measurement and treatment group	Baseline, T0 (*n* = 426)		Post-program, T1 (*n* = 276)		Six-months, T2 (*n* = 185)	
	Mean	95% CI	Mean	95% CI	Mean	95% CI
BMI z-score						
All	1.04	(0.93, 1.14)	1.02	(0.90, 1.15)	1.11	(0.97, 1.32)
Intervention	1.01	(0.87, 1.15)	0.98	(0.80, 1.15)	1.12	(0.91, 1.32)
Control	1.07	(0.92, 1.23)	1.07	(0.89, 1.26)	1.10	(0.91, 1.37)
*p*-value	0.5397		0.4448		0.9274	

Based on *t*-test.

Results based on linear mixed-effects indicate that the interaction term between group and time was statistically significant at 6 weeks post-program (*β* = −0.05, 95% CI, −0.09, −0.01, *p* = 0.023) (Table 3). Results based on the contrasts involving factor variables and their interactions show that, compared with the baseline values, BMI z-scores decreased at 6 weeks in the intervention group (−0.03, 95% CI, −0.066, −0.003; *p* = 0.032), but not in the control group. However, at six-months after the program, changes in BMI z-scores for both the intervention and control groups were not statistically significantly different from baseline (Table 4).

**Table 3:** Estimated parameters from repeated mixed-effects regressions on continuous BMI z-scores outcome measures.

	Estimate	95% CI	*p*-value
Fixed effects			
Intervention (ref = control)	−0.06	(−0.26, 0.14)	0.580
Time (ref = T0)			
T1	0.02	(−0.01, 0.04)	0.300
T2	0.00	(−0.06, 0.05)	0.886
Group x time			
Intervention x T1	−0.05	(−0.09, −0.01)	0.023
Intervention x T2	−0.01	(−0.08, 0.06)	0.858
Sex (ref = girls)			
Boys	0.18	(−0.02, 038)	0.073
Age (ref = 12+)			
Younger children (6–11 years)	−0.21	(−0.43, 0.00)	0.055
Site (ref = Illinois)			
California	−0.30	(−0.59, 0.01)	0.043
Iowa	0.23	(−0.06, 0.51)	0.114
Texas	0.12	(−0.18, 0.42)	0.429
Puerto Rico	−0.67	(−1.03, −0.31)	<0.001
Intercept	1.25	(0.94, 1.56)	<0.001
Random effects		Variance	
Intercept	1.03	(0.91, 1.18)	
Residual	0.02	(0.02, 0.03)	
Model fit		AIC	BIC
		1122.544	1,189.574

CI, Coinfidence intervals; AIC, Akaike information criterion; BIC, Bayesian information criterion.

**Table 4:** Changes in BMI z-scores at post-program (T1) and after 6-months (T2) from baseline.

Group	Change	Contrast	*p-*value	[95% confidence interval]	
All					
Intervention	T1 vs. baseline	−0.03	0.032	−0.066	−0.003
Control	T1 vs. baseline	0.02	0.300	−0.014	0.045
Intervention	T2 vs. baseline	−0.01	0.659	−0.057	0.036
Control	T2 vs. baseline	0.00	0.886	−0.058	0.050

Based on estimates from Table 3. BMI. Body mass index.

### 3.3. Analyses disaggregated by sex and age

In further analyses, we disaggregated the sample by age (young and teens) and sex. Results indicate that, for boys, there were no statistical differences between the control and intervention groups. For girls, the interaction term between group and time was statistically significant at 6 weeks post-program (*β* = −0.08, 95% CI, −0.13, −0.02, *p* = 0.007) (Supplementary Table S1a). Results based on the contrast show that, compared to the baseline, BMI z-scores for girls decreased at 6 weeks in the intervention group (−0.05, 95% CI, −0.095, −0.005, *p* = 0.03). In analyses disaggregated by age, results indicated that the intervention benefited the younger children, but not older children. The interaction term between group and time was statistically significant at 6 weeks post-program for younger children (*β* = −0.07, 95% CI, −0.12, −0.02, *p* = 0.003) (Supplementary Table S1b). Results based on the contrast show that, compared to the baseline, BMI z-scores for younger children decreased at 6 weeks in the intervention group (−0.04, 95% CI, −0.079, −0.011, *p* = 0.01).

### 3.4. Sensitivity analysis restricting to participants whose mothers were born in Mexico

In additional analyses where we restricted the sample to those whose mothers were born in Mexico; the conclusions remain. Meaning, that children in the intervention group observed a small decline in BMI z-scores at T1 (*β* = −0.05, 95% CI, −0.09, −0.01, *p* = 0.034), but not those in the control group. (Supplementary Table S1c).

### 3.5. Sensitivity analysis considering missing data

Given the proportion of missing data at the follow-up, we explored the data non-response pattern. We contrasted those with complete data for all time points (37%) with those missing at least one point time. Results from mixed-effects regressions, which included a dummy variable for incomplete *vs.* complete data, indicated that those who dropped out of the study did not differ in their baseline BMI z-scores. The remaining statistical inferences were unchanged. We further examined the data by analyzing only those with complete data, and the results remained unchanged.

## 4. Discussion

Despite decades of evidence-based recommendations and interventions, systematic reviews, consensus statements, with stakeholder and community members' advocacy, the effectiveness of childhood obesity prevention programs continues to be a (1, 12, 16, 20, 21, 24, 25, 31–34). The problem of obesity is particularly salient among Hispanic children and adolescents, accentuated by the burden of structural and social determinants of health, which limit access to opportunities and services that promote long-term engagement in health promotion behaviors (12, 35–37). Two recent systematic reviews on family-centered interventions for the treatment and prevention of childhood obesity found only a few articles with full methods and outcomes reporting effectiveness by using culturally related tools designed for US Hispanics (e.g., bilingual sessions) (16, 20). Few family-based or community interventions have an RCT design, and the reported outcomes of interventions are inconsistent, ranging from a slight decrease, no change, or a larger drop in BMI (particularly among those with higher BMI values at baseline) (16, 20).

Achieving BMI reduction in children and adolescents may be challenging due to their rapid physical growth in height and adiposity, particularly during early adolescence (14, 38). However, even smaller reductions of BMI-SDS have been associated with improved cardiometabolic health and body composition (11, 39). While participation in *Abriendo Caminos* was not associated with a large BMI reduction, participation was effective at reducing BMI z-scores at T1 by 0.03. Further, for girls, the reduction of BMI z-scores at T1 was 0.05 and for young children by 0.04. Although this is not a large difference, previous research has found that a reduction of BMI-z score of 0.09 or more has been associated with improved metabolic biomarkers in obese youth (39).

The objective of this primary prevention study was to prevent unhealthy weight gain, but 88% of parents and 54% of children who participated in the *Abriendo Caminos* program were already affected by overweight or obesity. However, weight loss was more common than weight gain in the intervention group. In particular, among those in the intervention group who were obese, 11% became overweight, and among those who were overweight, 15% became normal weight. In the control group, only 5% of those obese became overweight, and no one who was overweight became normal (Supplementary Table S2). The US Preventive Services Task Force (USPSTF) recommends 26 h or more of high-intensity clinical contact, in a period of 2 to 12 months, for successful weight loss (40), and there is no data on family-based preventive programs. Thus, the *Abriendo Caminos* program holds additional promise as reductions in BMI were observed, and not just maintenance of a healthy weight in the short-term, with 12 h of contact. Future goals include adding booster monthly sessions and including shorter and more frequent intervals for measurements to determine if the effects endure over a longer time with continued intervention. Offering culturally community-engaged, family-based programs is important for the sustainability of a healthy lifestyle, as stated in pediatric clinical practice guidelines (41).

There are several limitations and strengths to this study. The main limitation was the reduced number of participants who completed assessments at three-point times. Unfortunately, this is a common problem in health promotion programs designed and implemented among underserved and ethnically diverse groups. Immediate incentives, regular reminders, and engaging activities were integral elements of the program (22, 28). Retention was lower than we expected, due to the family/work demands and high mobility of the population. Sustainability is major barrier in pediatric weight management programs, attrition varies widely across clinical studies (4%–83%, median 37%) (42). In this community program, 70% and 47% completed the 6-week and six-months evaluations. As we recognize that Hispanic families have family and work demands, offering flexible times and multiple meeting options may help increase retention rates. Another major challenge we had with recruitment and retention of participants was due to climate and natural disasters we experienced during the course of the study, such as major hurricanes, flooding, earthquakes, and freezing. In the future, the efficacy of virtual delivery of the program is important to assess given the unpredictable challenges that can inevitably occur.

Additionally, *Abriendo Caminos* was only 6-weeks long, with 12 h of engagement, and longer term programs have shown greater efficacy. However, changes in BMI z-scores were observed within this time period, demonstrating that 6 weeks can be effective in decreasing excessive weight gain. Strengths include the cultural tailoring, multi-site, a 6-month follow-up to assess the sustainability of BMI changes, a theoretical underpinning, and inclusion of evidence-based curriculum with topics related to nutrition, screen time and sleep (22). Similarly, our whole family approach moves away from limiting program participation to parent-child dyads, thereby potentially expanding the program reach to multiple family members.

In conclusion, few family-based programs demonstrate an impact on primary outcomes. Even fewer are culturally-tailored and evaluated for long-term effects. *Abriendo Caminos* is a culturally-tailored whole family program coupled with cultural humility that considers elements of the Hispanic culture that facilitate healthy behavior change and effectively prevented unhealthy weight gain, as a first step. Families are active participants in the program, helping shape the program to meet their needs. Obesity poses a serious threat to Hispanic families, and efforts to reduce this risk are essential in slowing and preventing this public health crisis. Culturally-tailored programs, such as *Abriendo Caminos*, can become a staple in communities providing families with the knowledge to produce change and pathways to enact and sustain that change. Over time, these types of programs may help drive reductions in unhealthy weight gain and prevent high rates of chronic metabolic conditions within the Hispanic population in the future.

## Data Availability

The datasets presented in this article are not readily available because we obtained a certificate of confidentiality. Unidentified datasets, without location site, could be shared in the future. Requests to access the datasets should be directed to teranmd@illinois.edu.

## References

[1] Kris-Etherton PM Petersen KS Velarde G Barnard ND Miller M Ros E Barriers, opportunities, and challenges in addressing disparities in diet-related cardiovascular disease in the United States. J Am Heart Assoc. (2020) 9(7):e014433. 10.1161/JAHA.119.014433 32200727 PMC7428614

[2] Niles MT Bertmann F Belarmino EH Wentworth T Biehl E Neff R. The early food insecurity impacts of COVID-19. Nutrients. (2020) 12(7):2096. 10.3390/nu12072096 32679788 PMC7400862

[3] Beaunoyer E Dupéré S Guitton MJ. COVID-19 and digital inequalities: reciprocal impacts and mitigation strategies. Comput Human Behav. (2020) 111:106424. 10.1016/j.chb.2020.106424 32398890 PMC7213963

[4] Gassman-Pines A Ananat EO Fitz-Henley J. COVID-19 crisis impacts on parent and child psychological well-being. Pediatrics. (2020) 146(4):e2020007294. 10.1542/peds.2020-007294 32764151 PMC7546085

[5] Mozaffarian D Benjamin EJ Go AS Arnett DK Blaha MJ Cushman M Heart disease and stroke statistics--2015 update: a report from the American heart association. Circulation. (2015) 131(4):e29–e322. 10.1161/CIR.0000000000000152 25520374

[6] Schneiderman N Llabre M Cowie CC Barnhart J Carnethon M Gallo LC Prevalence of diabetes among hispanics/latinos from diverse backgrounds: the hispanic community health study/study of latinos (HCHS/SOL). Diabetes Care. (2014) 37(8):2233–9. 10.2337/dc13-2939 25061138 PMC4113173

[7] Ogden CL Fryar CD Martin CB Freedman DS Carroll MD Gu Q Trends in obesity prevalence by race and Hispanic origin-1999-2000 to 2017-2018. JAMA. (2020) 324(12):1208–10. 10.1001/jama.2020.14590 32857101 PMC7455882

[8] Di Angelantonio E Bhupathiraju SN Wormser D Gao P Kaptoge S de Gonzalez AB Body-Mass Index and all-cause mortality: individual-participant-data meta-analysis of 239 prospective studies in four continents. Lancet. (2016) 388(10046):776–86. 10.1016/S0140-6736(16)30175-1 27423262 PMC4995441

[9] Simmonds M Llewellyn A Owen CG Woolacott N. Predicting adult obesity from childhood obesity: a systematic review and meta-analysis. Obes Rev. (2016) 17(2):95–107. 10.1111/obr.12334 26696565

[10] Adab P Pallan MJ Lancashire ER Hemming K Frew E Griffin T A cluster-randomised controlled trial to assess the effectiveness and cost-effectiveness of a childhood obesity prevention programme delivered through schools, targeting 6-7 year old children: the WAVES study protocol. BMC Public Health. (2015) 15:488. 10.1186/s12889-015-1800-8 25968599 PMC4457211

[11] Reinehr T Lass N Toschke C Rothermel J Lanzinger S Holl RW. Which amount of BMI-SDS reduction is necessary to improve cardiovascular risk factors in overweight children? J Clin Endocrinol Metab. (2016) 101(8):3171–9. 10.1210/jc.2016-1885 27285295

[12] Ho M Garnett SP Baur L Burrows T Stewart L Neve M Effectiveness of lifestyle interventions in child obesity: systematic review with meta-analysis. Pediatrics. (2012) 130(6):e1647–71. 10.1542/peds.2012-1176 23166346

[13] Birch L Perry R Hunt LP Matson R Chong A Beynon R What change in body mass index is associated with improvement in percentage body fat in childhood obesity? A meta-regression. BMJ Open. (2019) 9(8):e028231. 10.1136/bmjopen-2018-028231 31473614 PMC6720247

[14] Brannsether B Eide GE Roelants M Bjerknes R Júlíusson PB. BMI And BMI SDS in childhood: annual increments and conditional change. Ann Hum Biol. (2017) 44(1):28–33. 10.3109/03014460.2016.1151933 26862707

[15] Skelton JA Buehler C Irby MB Grzywacz JG. Where are family theories in family-based obesity treatment?: conceptualizing the study of families in pediatric weight management. Int J Obes. (2012) 36(7):891–900. 10.1038/ijo.2012.56 PMC3977510 22531090

[16] Soltero EG Peña A Gonzalez V Hernandez E Mackey G Callender C Family-based obesity prevention interventions among hispanic children and families: a scoping review. Nutrients. (2021) 13(8):2690. 10.3390/nu13082690 34444850 PMC8402012

[17] Wilfley DE Fowler LA Hampl SE Dreyer Gillette ML Staiano AE Graham AK Implementation of a scalable family-based behavioral treatment for childhood obesity delivered through primary care clinics: description of the Missouri childhood obesity research demonstration study protocol. Child Obes. (2021) 17(S1):S39–S47. 10.1089/chi.2021.0175 34569843 PMC8575056

[18] Arredondo EM Ayala GX Soto S Slymen DJ Horton LA Parada H Latina mothers as agents of change in children’s eating habits: findings from the randomized controlled trial entre familia: reflejos de Salud. Int J Behav Nutr Phys Act. (2018) 15(1):95. 10.1186/s12966-018-0714-0 30285755 PMC6167856

[19] Hammons AJ Wiley AR Fiese BH Teran-Garcia M. Six-week Latino family prevention pilot program effectively promotes healthy behaviors and reduces obesogenic behaviors. J Nutr Educ Behav. (2013) 45(6):745–50. 10.1016/j.jneb.2013.01.023 23726891

[20] Tamayo MC Dobbs PD Pincu Y. Family-centered interventions for treatment and prevention of childhood obesity in Hispanic families: a systematic review. J Community Health. (2021) 46(3):635–43. 10.1007/s10900-020-00897-7 32734580

[21] Brown T Moore THM Hooper L Gao Y Zayegh A Ijaz S Interventions for preventing obesity in children. Cochrane Database Syst Rev. (2019) 7(7):CD001871. 10.1002/14651858.CD001871.pub4 31332776 PMC6646867

[22] Hannon BA Teran-Garcia M Nickols-Richardson SM Musaad SMA Villegas EM Hammons A Implementation and evaluation of the Abriendo Caminos program: a randomized control trial intervention for Hispanic children and families. J Nutr Educ Behav. (2019) 51(10):1211–9. 10.1016/j.jneb.2019.08.011 31706460

[23] Kerr ME Bowen M. Family evaluation: An approach based on Bowen theory. Co. WWN, editor (1988).

[24] Branscum P Sharma M. A systematic analysis of childhood obesity prevention interventions targeting Hispanic children: lessons learned from the previous decade. Obes Rev. (2011) 12(5):e151–8. 10.1111/j.1467-789X.2010.00809.x 20977600

[25] Tovar A Renzaho AN Guerrero A Mena N Ayala G. A systematic review of obesity prevention intervention studies among immigrant populations in the U.S. Curr Obes Rep. (2014) 3(2):206–22. 10.1007/s13679-014-0101-3 24818072 PMC4004797

[26] Child and Adolescent Health Measurement Initiative. 2020–2021 National Survey of Children’s Health (NSCH) data query. Data Resource Center for Child and Adolescent Health supported by the U.S. Department of Health and Human Services, Health Resources and Services Administration (HRSA), Maternal and Child Health Bureau (MCHB). Retrieved [03/25/23] from: www.childhealthdata.org

[27] CDC. A SAS Program for the 2000 CDC Growth Charts (ages 0 to <20 y) (2000). Available from: https://www.cdc.gov/nccdphp/dnpao/growthcharts/resources/sas.htm

[28] Barragan M Luna V Hammons AJ Olvera N Greder K Drumond Andrade FC Reducing obesogenic eating behaviors in Hispanic children through a family-based, culturally-tailored RCT: Abriendo Caminos. Int J Environ Res Public Health. (2022) 19(4):1917. 10.3390/ijerph19041917 35206123 PMC8872523

[29] McCulloch CE Searle SR. Generalized, Linear, and Mixed Models. John Wiley & Sons I, editor (2001).

[30] Molenberghs G Verbeke G. Linear mixed models for longitudinal data. 1st ed. New York, NY: Springer (2020).

[31] Daniels SR Hassink SG Committee On N. The role of the pediatrician in primary prevention of obesity. Pediatrics. (2015) 136(1):e275–92. 10.1542/peds.2015-1558 26122812

[32] Robert Wood Johnson Foundation. Maximizing the impact of obesity-prevention efforts in Latino communities: Key findings and strategic recommendations. (2015). Available from: https://www.tfah.org/report-details/the-state-of-obesity-2015/

[33] Stevens J Pratt C Boyington J Nelson C Truesdale KP Ward DS Multilevel interventions targeting obesity: research recommendations for vulnerable populations. Am J Prev Med. (2017) 52(1):115–24. 10.1016/j.amepre.2016.09.011 28340973 PMC5571824

[34] Sanyaolu A Okorie C Qi X Locke J Rehman S. Childhood and adolescent obesity in the United States: a public health concern. Glob Pediatr Health. (2019) 6:2333794X19891305. 10.1177/2333794X19891305 31832491 PMC6887808

[35] Sadeghi B Kaiser LL Hanbury MM Tseregounis IE Shaikh U Gomez-Camacho R A three-year multifaceted intervention to prevent obesity in children of Mexican-heritage. BMC Public Health. (2019) 19(1):582. 10.1186/s12889-019-6897-8 31096944 PMC6521467

[36] Otterbach L Mena NZ Greene G Redding CA De Groot A Tovar A. Community-based childhood obesity prevention intervention for parents improves health behaviors and food parenting practices among Hispanic, low-income parents. BMC Obes. (2018) 5:11. 10.1186/s40608-018-0188-2 29610670 PMC5870387

[37] Jones NL Breen N Das R Farhat T Palmer R. Cross-Cutting themes to advance the science of minority health and health disparities. Am J Public Health. (2019) 109(S1):S21–4. 10.2105/AJPH.2019.304950 30699031 PMC6356138

[38] Ohlsson C Bygdell M Nethander M Rosengren A Kindblom JM. BMI Change during puberty is an important determinant of adult type 2 diabetes risk in men. J Clin Endocrinol Metab. (2019) 104(5):1823–32. 10.1210/jc.2018-01339 30517677 PMC6456008

[39] Weiss R Shaw M Savoye M Caprio S. Obesity dynamics and cardiovascular risk factor stability in obese adolescents. Pediatr Diabetes. (2009) 10(6):360–7. 10.1111/j.1399-5448.2008.00504.x 19490496

[40] US Preventive Services Task Force Recommendation Statement, Grossman DC Bibbins-Domingo K Curry SJ Barry MJ Davidson KW Screening for obesity in children and adolescents: uSPSTF recommendation statement. JAMA. (2017) 317(23):2417–26. 10.1001/jama.2017.6803 28632874

[41] Styne DM Arslanian SA Connor EL Farooqi IS Murad MH Silverstein JH Pediatric obesity-assessment, treatment, and prevention: an endocrine society clinical practice guideline. J Clin Endocrinol Metab. (2017) 102(3):709–57. 10.1210/jc.2016-2573 28359099 PMC6283429

[42] Dhaliwal J Nosworthy NM Holt NL Zwaigenbaum L Avis JL Rasquinha A Attrition and the management of pediatric obesity: an integrative review. Child Obes. (2014) 10(6):461–73. 10.1089/chi.2014.0060 25496035

