# Supplementary material for: Randomized control trial of a childhood obesity prevention family-based program: “Abriendo Caminos” and effects on BMI
Source: Front Pediatr. 2023 Jun 7;11:1137825. doi: 10.3389/fped.2023.1137825 (PMC10282656; doi:10.3389/fped.2023.1137825)
Supplement: Supplementary file 1 [file Table1.pdf]

Supplementary Material

Randomized Control Trial of a Childhood Obesity Prevention Family-Based Program: "Abriendo Caminos" and Effects on BMI

Margarita Teran-Garcia[1], Amber J. Hammons[2], Norma Olvera[3], Kimberly Greder [4], Maria Plaza-Delrestre[5], Flavia Cristina Drumond Andrade[6], Barbara Fiese[7], Angela Wiley[8], and the Abriendo Caminos Research Team

*** Correspondence:** Margarita Teran-Garcia, teranmd@illinois.edu

1 Supplementary Tables

Table S1a. Estimated parameters from repeated mixed-effects regressions on continuous BMI z-scores outcome measures. Analyses of disaggregated data for boys and girls, controlled for age.

	Boys			Girls		
Fixed effects	Estimate	95% CI	*p-value*	Estimate	95% CI	*p-value*
Intervention (ref=control)	0.117	(-0.188,0.423)	0.452	-0.197	(-0.46,0.065)	0.141
Time (ref=T0)						
T1	0.001	(-0.048,0.05)	0.965	0.027	(-0.006,0.061)	0.113
T2	-0.003	(-0.087,0.08)	0.943	-0.004	(-0.074,0.065)	0.904
Group x time						
Intervention x T1	-0.017	(-0.083,0.048)	0.604	-0.077	(-0.133,-0.021)	0.007
Intervention x T2	-0.014	(-0.118,0.091)	0.798	-0.002	(-0.098,0.094)	0.965
Age (ref=12+)						
Younger children (6-11 years)	-0.081	(-0.439,0.277)	0.658	-0.306	(-0.576,-0.037)	0.026
Site (ref=Illinois)						
California	-0.408	(-0.838,0.022)	0.063	-0.263	(-0.662,0.136)	0.197
Iowa	0.307	(-0.117,0.731)	0.156	0.166	(-0.227,0.558)	0.407
Texas	0.231	(-0.196,0.658)	0.289	-0.025	(-0.453,0.404)	0.909
Puerto Rico	-0.517	(-1.101,0.067)	0.083	-0.828	(-1.271,-0.386)	0.000
Intercept	1.195	(0.773,1.617)	0.000	1.448	(1.015,1.881)	0.000
Random effects		Variance			Variance	
Intercept	1.0666	(0.869,1.309)		0.9746	(0.821,1.157)	
Residual	0.0227	(0.017,0.03)		0.0252	(0.018,0.035)	
Model fit		AIC	BIC		AIC	BIC
		504.96	556.65		632.10	686.70

AIC = Akaike information criterion, BIC = Bayesian information criterion

Table S1b. Estimated parameters from repeated mixed-effects regressions on continuous BMI z-scores outcome measures. Analyses of disaggregated data for young or old children controlled for sex.

	Younger			Older		
Fixed effects	Estimate	95% CI	*p-value*	Estimate	95% CI	*p-value*
Intervention (ref=control)	0.015	(-0.225,0.256)	0.900	-0.328	(-0.685,0.029)	0.072
Time (ref=T0)						
T1	0.029	(-0.007,0.065)	0.113	-0.021	(-0.066,0.024)	0.358
T2	0.002	(-0.058,0.062)	0.958	-0.018	(-0.137,0.101)	0.763
Group x time						
Intervention x T1	-0.07	(-0.124,-0.024)	0.003	0.052	(-0.043,0.146)	0.284
Intervention x T2	-0.01	(-0.085,0.072)	0.874	-0.014	(-0.177,0.149)	0.870
Sex (ref=Girls)						
Boys	0.25	(0.027,0.482)	0.028	-0.002	(-0.377,0.372)	0.990
Site (ref=Illinois)						
California	-0.28	(-0.61,0.055)	0.102	-0.353	(-0.949,0.243)	0.246
Iowa	0.23	(-0.09,0.541)	0.161	0.204	(-0.442,0.85)	0.536
Texas	0.03	(-0.333,0.384)	0.888	0.496	(-0.053,1.046)	0.077
Puerto Rico	-0.72	(-1.137,-0.3)	0.001	-0.525	(-1.22,0.17)	0.139
Intercept	0.98	(0.679,1.283)	0.000	1.369	(0.809,1.928)	0.000
Random effects		Variance			Variance	
Intercept	1.0620	(0.916,1.231)		0.8881	(0.684,1.153)	
Residual	0.0245	(0.019,0.032)		0.0217	(0.014,0.034)	
Model fit		AIC	BIC		AIC	BIC
		848.49	907.45		285.35	328.09

AIC = Akaike information criterion, BIC = Bayesian information criterion

Table S1c. Estimated parameters from repeated mixed-effects regressions on continuous BMI z-scores outcome measures restricted to participants whose mothers are from Mexico.

	Mexican mothers		
Fixed effects	Estimate	95% CI	*p-value*
Intervention (ref=control)	0.024	(-0.212, 0.26)	0.841
Time (ref=T0)			
T1	0.027	(-0.007, 0.061)	0.114
T2	-0.010	(-0.069, 0.048)	0.727
Group x time			
Intervention x T1	-0.049	(-0.094, -0.004)	0.034
Intervention x T2	0.004	(-0.075, 0.082)	0.929
Sex (ref=Girls)			
Boys	0.083	(-0.146, 0.313)	0.478
Age (ref=Older)			
Young	-0.172	(-0.422, 0.078)	0.177
Site (ref=Illinois)			
California	-0.203	(-0.516, 0.109)	0.203
Iowa	0.263	(-0.049, 0.575)	0.099
Texas	0.154	(-0.193, 0.502)	0.384
Intercept	1.173	(0.82, 1.527)	0.000
Random effects		Variance	
Intercept	0.9873	(0.838, 1.163)	
Residual	0.0207	(0.017, 0.026)	
Model fit		AIC	BIC
		720.97	779.03

AIC = Akaike information criterion, BIC = Bayesian information criterion

Table S2. Changes in weight status (wt_status) categories according to the intervention

Intervention		T1_wtstatus: Normal	Obese	Overwt	Underwt	Total	
T0_wtstatus	Normal	70	0	2	0	72	n
		**97.2**	**0.0**	**2.8**	**0.0**	**100.0**	%
	Obese	0	49	6	0	55	n
		**0.0**	**89.1**	**10.9**	**0.0**	**100.0**	%
	Overwt	4	2	21	0	27	n
		**14.8**	**7.4**	**77.8**	**0.0**	**100.0**	%
	Underwt	1	0	0	3	4	n
		**25.0**	**0.0**	**0.0**	**75.0**	**100.0**	%
	Total	75	51	29	3	158	n
		**47.5**	**32.3**	**18.4**	**1.9**	**100.0**	%

Control		T1_wtstatus: Normal	Obese	Overwt	Total	
T0_wtstatus	Normal	49	0	2	51	n
		**97.2**	**0.0**	**3.9**	**100.0**	%
	Obese	0	41	2	43	n
		**0.0**	**95.4**	**4.7**	**100.0**	%
	Overwt	0	0	23	23	n
		**0.0**	**0.0**	**100.0**	**100.0**	%
	Underwt	1	0	0	1	n
		**100.0**	**0.0**	**0.0**	**100.0**	%
	Total	50	41	27	118	n
		**42.4**	**34.8**	**22.9**	**100.0**	%

Most children remained in the same categories between T0 and T1 in both *Abriendo Caminos* intervention and control groups. However, weight loss was more common than weight gain in the intervention group. In particular, among those in the intervention group who were obese, 11% became overweight, and among those who were overweight, 15% became normal weight. In the control group, only 5% of those obese became overweight, and no one who was overweight became normal.
